# Intervention Mechanisms of Xinmailong Injection, a* Periplaneta Americana* Extract, on Cardiovascular Disease: A Systematic Review of Basic Researches

**DOI:** 10.1155/2019/8512405

**Published:** 2019-07-22

**Authors:** Shan-Shan Lin, Chun-Xiang Liu, Xian-Liang Wang, Jing-Yuan Mao

**Affiliations:** ^1^Cardiovascular Department, First Teaching Hospital of Tianjin University of Traditional Chinese Medicine, Tianjin 300381, China; ^2^Evidence-Based Medicine Center, Tianjin University of Traditional Chinese Medicine, Tianjin 301617, China

## Abstract

**Background:**

At present, the prevention and treatment of cardiovascular disease in the world are facing severe challenges. Xinmailong injection, which is derived from the animal medicine* Periplaneta Americana*, has certain advantages in the clinical treatment of cardiovascular disease. This study systematically evaluated the basic research reports of Xinmailong Injection on cardiovascular disease and made its pharmacological mechanisms more clear.

**Methods:**

Basic research reports on the intervention mechanisms of Xinmailong Injection on cardiovascular disease in PubMed, EMBASE, Cochrane Library (No. 2, 2019), CNKI, Wan Fang, and VIP databases were searched. The search time limit was from the establishment of the database to February 2019. The literature was screened according to inclusion and exclusion criteria, and then the data were extracted and a descriptive analysis of the pharmacological mechanisms of Xinmailong Injection on cardiovascular disease was performed.

**Results:**

Finally, twenty-two basic research reports were included. The intervention mechanisms of Xinmailong Injection on cardiovascular disease mainly includes the following: inhibiting oxidative stress and inflammatory reaction; regulating autophagy; promoting Ca^2+^ influx by activating excitability of excitation-contraction coupling (ECC); inhibiting overexpressions of transforming growth factor-*β*1 (TGF-*β*1) and connective tissue growth factor (CTGF) to regulate the dynamic balance of matrix metalloproteinases (MMPs) and tissue inhibitors of matrix metalloproteinases (TIMPs); inhibiting the phosphorylation of extracellular regulated protein kinases 1/2 (ERK1/2), protein kinase B (AKT), and glycogen synthase kinase 3*β* (GSK3*β*) proteins and overexpression of the downstream transcription factor GATA4 in the nucleus; regulating vascular endothelial factors and so on.

**Conclusions:**

Xinmailong Injection can protect cardiomyocytes and maintain the normal function of the heart in various ways, thus effectively preventing the development of cardiovascular disease. Therefore, Xinmailong Injection has great potential for clinical application, and more basic researches need to be carried out to explore the medicinal value of Xinmailong Injection.

## 1. Background

Cardiovascular disease is currently the first cause of death in the world. According to the “China Cardiovascular Disease Report 2017” [1], the number of people suffering from cardiovascular disease in China is about 290 million. At present, the prevention and treatment of cardiovascular disease have achieved initial results, but they still face serious challenges. Animal material medicine is an important part of traditional medicinal resources. A large number of animal material medicine have been developed as important raw materials for modern Chinese patent medicines and western medicines due to their special biological activities.* Periplaneta Americana* is part of them. Due to its rich source and low cost, the medicinal value of* P. Americana* has enormous development potential. Since the 1980s, Chinese pharmacologist Professor Li Shunan has developed a series of new drugs, such as Kangfuxin Liquid, Xinmailong Injection, and Ganlong Capsule, using the* P. Americana* as raw material. They are used in many fields such as fighting tumors, improving heart function, inhibiting liver fibrosis, and protecting the gastrointestinal mucosa [2]. Among them, Xinmailong Injection is mainly an auxiliary drug for chronic heart failure (CHF), and its main active ingredients are adenosine, inosine, protocatechuic acid, and pyroglutamate dipeptides [3–5]. Xinmailong Injection became the second class of traditional Chinese medicines in the national new drugs in 2006, which was approved by the State Food and Drug Administration (SFDA). Its efficacy in treating cardiovascular disease has been highly recognized since its launch. It has been recommended by many expert consensus and guidelines such as “Expert Consensus on Diagnosis and Treatment of Chronic Heart Failure by Integrative Chinese and Western Medicine” [4] and “Guidelines for the Diagnosis and Treatment of Acute Myocardial Infarction by Integrative Chinese and Western Medicine” [6].

In this study, the basic researches about the intervention mechanisms of Xinmailong Injection on cardiovascular disease were systematically organized. The aim is to provide a reference for further research and clinical application of Xinmailong Injection.

## 2. Methods

### 2.1. Inclusion Criteria

The inclusion criteria are the basic researches about the intervention mechanisms of Xinmailong Injection on cardiovascular disease. Experimental models include animals, organs, tissues, and cells associated with cardiovascular disease.

### 2.2. Exclusion Criteria

Exclusion criteria were (1) to observe the comprehensive efficacy of Xinmailong Injection combined with other interventions; (2) for the repeated publication of the literature, excluding low-quality literature; (3) the full text not obtained; (4) clinical research, case reports, expert consensus, review, systematic review, and meta-analysis.

### 2.3. Search Strategy

The basic researches about the intervention mechanisms of Xinmailong Injection on cardiovascular disease in PubMed, EMBASE, Cochrane Library (No. 2 of 2019), CNKI, Wan Fang, and VIP databases were searched. The search time was from the database establishment to February 2019. Search terms included* P. Americana*, American cockroach, Xinmailong, heart failure, ventricular dysfunction, blood vessel, artery, heart, cardiac, cardiovascular, myocardium, myocardial, and the like. The search strategy was a combination of subject words and free words. In addition, we screened the reference list of relevant literature to avoid omissions. Take PubMed as an example. The specific search strategy is shown in Table 1.

### 2.4. Literature Screening and Data Extraction

Literature screening and data extraction were performed according to the inclusion and exclusion criteria. The data extraction includes title, author, publication year, experimental model, observation indicators, and research result, etc. Considering this article is a summary of the intervention mechanisms of Xinmailong Injection on cardiovascular disease, a descriptive analysis was performed.

## 3. Results

### 3.1. Literature Screening Process and Results

**Figure 1:**
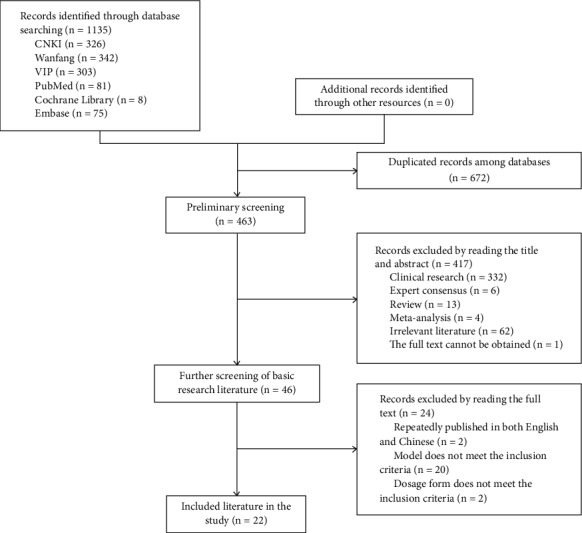
Flowchart of the literature selection process.

A total of 1135 pieces of literature were retrieved and 46 pieces of possible related literature were screened out. After reading the full text, twenty-two pieces of literature were finally included. The literature screening process and results are shown in Figure 1.

### 3.2. Literature Overview

The included literature consists of 4 pieces of English literature and 18 Chinese literature. The literature was published in 1995~2019. The literature published in the past five years accounted for 45.45% (10/22). Experimental models included animals with cardiovascular disease (such as myocardial cell injury, myocardial ischemia-reperfusion injury, myocardial infarction, atherosclerosis, cardiomyopathy, heart failure, or pulmonary hypertension, etc.), isolated rat heart, H9C2 cells, and hypoxic-reoxygenated rat cardiomyocytes, and the like. See Table 2 for details.

### 3.3. Intervention Mechanism of Xinmailong Injection on Cardiovascular Disease

The main indication for Xinmailong Injection is CHF, which is the terminal stage of the progressive development of various cardiovascular diseases. The early manifestations of heart failure (HF) are myocardial cell damage, hypertrophy, and necrosis, followed by myocardial fibrosis (MF) and ventricular enlargement. Therefore, Xinmailong Injection can also be widely used in the treatment of other cardiovascular diseases. Current researches show that Xinmailong Injection mainly interferes with the cardiovascular system through the following various mechanisms.

#### 3.3.1. Protective Mechanism of Xinmailong Injection on Cardiomyocytes

Studies have shown that myocardial cell damage is closely related to Oxidative Stress (OS) and inflammation [7]. OS refers to an unbalanced state caused by excessive oxidation of the body. In this state, the body's ability to synthesize reactive oxygen species (ROS) increases and the ability to resist oxidation decreases, resulting in damage to the body's tissue function. When OS occurs, the levels of lipid peroxides and ROS are significantly increased. The increase of ROS levels can further increase the expression of proinflammatory factors by activating nuclear factor kappa B (NF-KB) system, thereby promoting the inflammatory response [8, 9].

Wu Jianxin et al. [10] found that Xinmailong Injection can significantly reduce the J-point displacement (increased or lowered) on the electrocardiogram (ECG) of rabbits caused by isoproterenol so that the J-point position is close to the normal equipotential line. This suggests that Xinmailong Injection has a protective effect on isoproterenol-induced myocardial ischemic injury. Xinmailong Injection can also significantly reduce the frequency of ischemic arrhythmias in rabbits. Studies [11] on the rats further confirmed that Xinmailong Injection can correct the J-point displacement on the ECG caused by myocardial ischemia, and Xinmailong Injection can significantly reduce the level of the lipid peroxide malondialdehyde (MDA), reduce the activity of creatine phosphokinase (CPK) and lactate dehydrogenase (LDH) in blood, and improve glutathione peroxidase (GSH-PX) and SOD activity in myocardial tissue. Tian Kunlun and Jiang Yu [12] found that Xinmailong Injection can increase SOD activity and NO_2_^−^/NO_3_^−^ (ratio of nitrite to nitrate) level, correct acidosis, improve circulating blood flow, and increase urine output in rabbits of ischemia-reperfusion injury induced by hypovolemic shock. Li Zhengtao et al. [13] found in the cell experiment that Xinmailong Injection can significantly reduce the production of ROS in H9C2 cells and increase the expression of antioxidant enzymes such as superoxide dismutase- (SOD-) 1, SOD-2, and heme oxygenase (HO)-1, which indicates that the protective effect of Xinmailong Injection on cardiomyocytes is achieved by anti-lipid peroxidation and inhibition of OS.

By observing the protective effect of Xinmailong Injection on myocardial ischemia-reperfusion injury in young rabbits, Cao Hongxiao et al. [14] found that Xinmailong Injection can significantly inhibit the levels of endothelial constitutive nitric oxide synthase (ecNOS), MDA, and creatine kinase (CK) and the infiltration of inflammatory cells after reperfusion. Therefore, the researchers believe that Xinmailong Injection reduces the production of oxygen free radicals by anti-lipid peroxidation, which inhibits the inflammatory response, thereby reducing the damage of vascular endothelial cells and cardiomyocytes caused by reperfusion. Zhang Lijuan et al. [15] found that the levels of serum CK-MB, myocardial NF-*κ*B, and TNF-*α* reached a peak in neonatal rats after 6 hours of asphyxia. Moreover, the expression of NF-*κ*B and TNF-*α* was positively correlated (r=0.979, P<0.01). Xinmailong Injection can significantly inhibit the above changes. This suggests that Xinmailong Injection can reduce the expression of proinflammatory factors by inhibiting the activation of the NF-kB system, thereby protecting the myocardial injury. Xinmailong Injection can significantly inhibit the above myocardial damage. Combined with previous studies, it can be inferred that Xinmailong Injection can reduce the expression of proinflammatory factors by inhibiting the activation of the NF-kB system.

#### 3.3.2. Defensive Mechanism of Xinmailong Injection on Drug Cardiotoxicity

Autophagy refers to the process by which cells use lysosomes to degrade their damaged organelles and macromolecules. This process is regulated by the autophagy-related gene (Atg). The signaling pathways that regulate autophagy are complex. These signaling pathways interact with each other and form a huge regulatory network. Studies have shown that ROS is one of the major intracellular signal transducers that maintain autophagy [31]. At the same time, autophagy, as a protective and defense mechanism widely present in cells, plays an important role in alleviating ROS-mediated cell damage [32].

Liu Wei et al. [16] found that long-term use of doxorubicin significantly reduced the SOD activity and increased the MDA level in rats. While taking doxorubicin, loading and using Xinmailong Injection can increase the SOD activity and MDA level slightly. The results indicate that Xinmailong Injection can alleviate the cardiotoxicity of anthracyclines by activating the endogenous ROS scavenging system. Xinmailong Injection has the same preventive effect on myocardial toxicity induced by doxorubicin and epirubicin [17, 18]. Yuan Lili and Yang Zhihua [19] observed that the activity of H9C2 cells induced by doxorubicin was significantly decreased, the level of SOD in cells decreased and the level of MDA increased, the activity and expression of Caspase-3 in cells increased, and the accumulation of autophagy-related protein LC3B increased. The Xinmailong Injection can obviously reduce the cardiotoxicity induced by doxorubicin. This study suggests that the protective effect of Xinmailong Injection is related to the regulation of autophagy.

Li Hui et al. [20] conducted the autophagy-related studies and found that the increase in PI3K/AKT levels and the inhibition of P38MAPK and ERK1/2 phosphorylation contribute to the enhancement of anti-autophagy activity of Xinmailong Injection. Therefore, the researchers believed that Xinmailong Injection can inhibit autophagy by activating PI3K/AKT signaling pathway and inhibiting ERK1/2 and P38 MAPK signaling pathways, which is an important mechanism for Xinmailong Injection to reduce the cardiotoxicity of drugs.

Li Jie et al. [21] found that Xinmailong Injection significantly inhibited the upregulation of interleukin- (IL-) 1b, IL-6, and tumor necrosis factor- (TNF-) *α* levels and the decrease of LC3 release in H9C2 cells. However, this effect was inhibited by mitochondrial autophagy inhibitor Mdivi-1 and autophagy-related protein Atg7 siRNA. The study also found that Xinmailong Injection significantly inhibited the expression of PINK1, Parkin, Nix, and Beclin-1 and promoted the expression of Mitofusin1, Mitofusin2, Opa1, Drp1, and P62. This suggests that Xinmailong Injection can reduce the cardiotoxicity of the drug by regulating autophagy mediated by the PINK1/Parkin signaling pathway.

#### 3.3.3. Mechanism of Xinmailong Injection Enhancing Myocardial Contractility

Studies have shown that the enhancement of myocardial contractility can be achieved by activating myocardial excitation-contraction coupling (ECC) excitability to promote Ca^2+^ influx in cardiomyocytes. ECC refers to the signal transduction process of cell membrane depolarization mediated by Ca^2+^. When ECC excitability is activated, the Ca^2+^ on the cell membrane is transiently increased. At this time, Ca^2+^ and troponin are combined, and the transverse bridge on the thick muscle wire is combined with the thin filament to oscillate, thereby causing the myocardial cells to contract. The transport stability of Ca^2+^ is achieved by adjusting the voltage-dependent Ca^2+^ channel, Ca^2+^-ATPase, and Na^+^/Ca^2+^ exchanger.

Peng Fang et al. [22, 23] conducted multiple studies and found that Xinmailong Injection can promote Ca^2+^ influx in rat cardiomyocytes, and this effect cannot be inhibited by verapamil, a Ca^2+^ channel blocker. Therefore, it is concluded that Xinmailong Injection does not promote Ca^2+^ influx through Ca^2+^ channels. However, a further study [13] by Li Zhengtao et al. using Ca^2+^ imaging technology in H9C2 cells revealed that Xinmailong Injection can be inhibited by ML218-HCl (a T-type Ca^2+^ channels antagonist instead of L-type Ca^2+^ channel). Therefore, it is believed that Xinmailong Injection can increase cellular Ca^2+^ influx by activating T-type Ca^2+^ channels. Meanwhile, Li Zhengtao et al. also observed that Xinmailong Injection can inhibit Na^+^/K^+^-adenosine triphosphate (ATP) enzyme activity. This process can reduce the transmembrane electrochemical gradient of Na^+^, weaken the Na^+^/Ca^2+^ exchange capacity, reduce the Ca^2+^ export, and increase the concentration of Ca^2+^ in the myocardial cells, thereby enhancing the myocardial contractility.

#### 3.3.4. Mechanism of Xinmailong Injection Inhibiting MF

MF is a pathological phenomenon in which cardiac fibroblasts (CFs) abnormally proliferate and collagen deposition in the extracellular matrix (ECM), imbalance of various types of collagen, and disordered collagen arrangement are observed [33]. This process is one of the main causes of ventricular remodeling and cardiac ejection disorders [34, 35].

Transforming growth factor-*β* (TGF-*β*) is an important cytokine during MF [36]. It promotes the differentiation of resting fibroblasts into CFs. CFs can synthesize and secrete large amounts of collagen and can also produce matrix metalloproteinases (MMPs) and tissue inhibitors of matrix metalloproteinase (TIMPs) [37]. MMPs are a group of Zn^2-^ dependent proteolytic enzymes that specifically and efficiently degrade ECM. TIMPs can maintain the normal structure and function of ECM by regulating the activity of MMPs. Under physiological conditions, the expression of MMPs was extremely low; under pathological conditions, MMPs expression was significantly upregulated. Studies have shown that the occurrence of MF is related to the upregulation of MMP-2 and MMP-9 expression [38]. Li Hui et al. [20] found that Xinmailong Injection can reduce the levels of TGF-*β*1 mRNA and MMP-9 in the serum of rats with HF. Clinical studies have also confirmed that Xinmailong Injection can inhibit the upregulation of MMP-1 and MMP-9 expression in serum of HF patients [39, 40] and reduce ECM collagen deposition, thereby counteracting MF.

Connective tissue growth factor (CTGF) is a downstream factor specifically induced by TGF-*β*. It mediates the profibrotic progression of TGF-*β* and is an excellent marker of MF. The expression of TGF-*β* and CTGF increased synchronously during fibrosis, which together promoted ECM collagen deposition [41, 42]. Liu Guohong et al. [24] found that Xinmailong Injection can significantly inhibit the expression of CTGF and decrease the deposition of ECM collagen in cardiomyocytes of rats with alcoholic cardiomyopathy, indicating that Xinmailong Injection can effectively delay the process of MF.

#### 3.3.5. Mechanism of Xinmailong Injection Delaying the Development of Cardiac Hypertrophy

Cardiac hypertrophy is a complex dynamic process that is regulated by multiple factors. Its early change is an adaptive compensatory response. This reaction is caused by pathological factors such as excessive pressure or volume overload. With the long-term existence of pathological stimuli, compensatory cardiac hypertrophy may eventually develop into CHF.

Qi Jianyong et al. [3] found that Xinmailong Injection can effectively improve cardiac structure and function abnormalities in mice with transverse aortic coarctation (TAC) surgery caused by pressure overload and reduce ejection fraction and the thickness of the diastolic left ventricular posterior wall (LVPW). The downstream transcription factor GATA4 is a nuclear transcription factor closely related to cardiac development and plays a key role in the development of cardiomyocyte differentiation, cardiac hypertrophy, and HF [43]. Qi Jianyong et al. further carried out experiments in H9C2 cells and found that Xinmailong Injection can inhibit the phosphorylation of ERK1/2, AKT, and glycogen synthase kinase 3*β* (GSK3*β*) associated with cardiac hypertrophy and further inhibit the overexpression of the low downstream factor GATA4 in the nucleus. Therefore, this study supported that Xinmailong Injection can prevent myocardial decompensated hypertrophy from its inhibition of phosphorylation of ERK1/2, AKT/GSK3*β* pathway, and inhibition of overexpression of GATA4.

#### 3.3.6. Protective Mechanism of Xinmailong Injection on Vascular Structure and Function

Vascular endothelial cells are located between plasma and vascular tissue. It not only completes the exchange of metabolites of plasma and tissue fluids but also synthesizes and secretes a variety of biologically active substances. Bioactive substances can maintain the normal contraction and relaxation of blood vessels, regulate blood pressure (BP), and balance blood coagulation and hemolysis.

Endothelin (ET) and nitric oxide (NO) are a major endothelium-dependent relaxation and contraction vascular substance. ET has the functions of contracting blood vessels, promoting platelet adhesion and aggregation, and damaging the vascular endothelium. NO has the functions of dilating blood vessels, inhibiting blood cell adhesion, and anti-vascular smooth muscle cell proliferation. Wu Jianxin et al. [25] observed the effect of Xinmailong Injection on pulmonary hypertension induced by MCT and found that Xinmailong Injection can significantly reduce pulmonary artery pressure (PAP) and increase plasma NO level and ET level. This indicates that Xinmailong Injection can reduce pulmonary hypertension by regulating vascular endothelial factor.

The expression of endothelin-1 (ET-1) and myocardial hypoxia-inducible factor-1*α* (HIF-1*α*) can reflect the hypoxic state of the body. Huang Lixin and Wu Xingheng [26] found that the expression of HIF-1*α*, ET-1, and CK was significantly increased in neonatal rats with asphyxia, and the expression of HIF-1*α* was positively correlated with plasma ET-1 level (r = 0. 876, P < 0.01). Xinmailong Injection can effectively inhibit the expression of HIF-1*α* in the myocardium and decrease the levels of ET-1 and CK in plasma. This indicates that Xinmailong Injection can improve the myocardial ischemia and hypoxia injury by regulating vascular endothelial factor, making the blood vessels patency and blood flow normal, so as to ensure sufficient blood and oxygen supply to the myocardium.

Prostacyclin (PGI2) is an important vasodilator secreted by vascular endothelial cells, and its stable metabolite is 6-keto-prostaglandin F1a (6-Keto-PGF1a). By observing the intervention effect of Xinmailong Injection on vascular endothelial cells in rats with arteriosclerosis, Zhang Wenjing et al. [27] found that Xinmailong Injection can significantly reduce plasma ET levels and increase serum NO levels and plasma 6-Keto-PGF1a levels. In addition, Sun Lin and Zhang Wenjing [28] also found that Xinmailong Injection can significantly reduce total cholesterol (TC), triglyceride (TG), and low-density lipoprotein cholesterol (LDL-C) levels and raise high-density lipoprotein cholesterol (HDL-C) level in the serum of atherosclerotic (AS) rats. It is suggested that Xinmailong Injection can prevent and delay the progression of AS by regulating vascular endothelial factor and improving blood lipid status.

#### 3.3.7. Mechanism of Antiarrhythmia of Xinmailong Injection

Wu Jianxin et al. [29] found that pretreatment of healthy rabbits with an Injection of Xinmailong Injection through the ear vein can significantly prolong the survival time of rabbits during continuous perfusion of BaCl_2_ solution and reduce the arrhythmia caused by a bolus injection of BaCl_2_ solution. For rabbit models with arrhythmia, Xinmailong Injection can significantly improve the rate of sinus arrhythmia conversion. Arrhythmias such as atrial fibrillation or rapid atrial stimulation can cause a shortening of the atrial effective refractory period (AERP) [44]. Li Fengde et al. [44] found that continuous injection of 5-day Xinmailong Injection for rabbits can effectively prevent the shortening of AERP200 and AERP150 caused by rapid pacing for 6 hours. This indicates that Xinmailong Injection can effectively prevent atrial electrical remodeling. Li Hui et al. [20] found that Xinmailong Injection can improve the prolongation of QT and QTc on electrocardiogram induced by epirubicin. In addition, prolongation of QT dispersion (QTd) in patients with ischemic heart disease can predict the occurrence of malignant arrhythmia and sudden death. Clinical researches found that Xinmailong Injection can reduce the prolongation of QTd and prevent the occurrence of arrhythmia.

The researchers considered that the antiarrhythmia function of Xinmailong Injection may be related to the following mechanisms: (1) by regulating the activity of channel proteins transporting Na^+^, Ca^2+^, and K^+^ on the cardiomyocyte membrane to ensure the normal cardiac electrical conduction pathway and (2) by improving myocardial cell metabolism and scavenging oxygen free radicals to protect the integrity of myocardial cell membrane structure and function, thereby maintaining the stability of a variety of ion channels. At present, the specific mechanism of antiarrhythmia of Xinmailong Injection is still unclear and needs further exploration [45].

## 4. Discussion

### 4.1. Intervention Mechanism of Xinmailong Injection on Cardiovascular Disease

This study shows that the intervention mechanism of Xinmailong Injection on cardiovascular disease mainly includes the following points: (1) Xinmailong Injection inhibits OS by inhibiting the synthesis of ROS and promoting the activity of antioxidant enzymes and reduces the expression of proinflammatory factors by inhibiting the activation of NF-kB system. Therefore, the damage, degeneration, and necrosis of cardiomyocytes are alleviated. (2) Xinmailong Injection protects cardiomyocytes from drug toxicity by activating endogenous ROS clearance systems and regulating cellular autophagy. (3) Xinmailong Injection enhances myocardial contractility by activating ECC excitability and promoting Ca^2+^ influx. Maintaining the normal transport of Na^+^, Ca^2+^, and K^+^ on the myocardial cell membrane may also be an important mechanism of antiarrhythmia of Xinmailong Injection. (4) Xinmailong Injection regulates the dynamic balance of MMPs and TIMPs by inhibiting the overexpression of TGF-*β*1 and CTGF, thereby maintaining the normal structure and function of ECM, inhibiting collagen deposition and MF. (5) Xinmailong Injection delays the development of cardiac hypertrophy by inhibiting the phosphorylation of extracellular regulated protein kinases 1/2, AKT, and GSK3*β* proteins and overexpression of the downstream factor GATA4 in the nucleus. (6) Xinmailong Injection protects the structure and function of blood vessels by regulating vascular endothelial factor disorder and improving blood lipid status so that the heart muscle can obtain sufficient blood and oxygen supply. (7) The mechanism of antiarrhythmia of Xinmailong Injection is not clear. This may be related to its regulation of the activity of ion transport pathway proteins on the cell membrane to maintain the normal transport of Na^+^, Ca^2+^, and K^+^ on the myocardial cell membrane. This may also be related to its ability to scavenge oxygen free radicals to protect cell membrane structure and function.

Excessive activation of the neuroendocrine system is considered to be a key process leading to HF. Among them, the renin-angiotensin-aldosterone system (RAAS) plays a major role and can affect multiple links of the above mechanisms. Clinical studies have found that Xinmailong Injection can inhibit the excitability of the RAAS system. This may be a key intervention mechanism of Xinmailong Injection on cardiovascular disease. It is expected to carry out a large number of relevant basic researches for further exploration.

### 4.2. Clinical Application Value of Xinmailong Injection in the Treatment of HF

The current conventional drugs for treating HF have certain deficiencies while improving symptoms. Diuretics can cause electrolyte imbalance and hypotension. Beta blockers have a significant negative inotropic effect on the heart and may cause bradycardia or conduction block. Digitalis drugs may cause poisoning due to the narrow treatment width. Meta-analyses [46–48] showed that Xinmailong Injection can significantly improve a variety of clinical indicators related to cardiac function, thereby improving the clinical efficacy of patients with HF. In addition, Xinmailong Injection had the effects of regulating BP, enhancing myocardial contractility, and preventing arrhythmia and thus can prevent the occurrence of the above adverse reactions to a certain extent. However, Xinmailong Injection combined with conventional treatment of western medicine may cause adverse skin reactions [RR=2.04, 95% CI (1.05, 3.96), P=0.03] [49]. In addition, the clinical cost-effectiveness analysis of Xinmailong Injection in the treatment of HF showed that, compared with the conventional treatment group, the combination of Xinmailong and conventional treatment group had low cost, high efficiency, and better economic benefits. In summary, Xinmailong Injection plays an important role in improving the quality of life of patients and reducing the medical burden on families and society. Its medicinal value has great potential for development.

### 4.3. The Direction and Thinking of the Future Research of Xinmailong Injection

Xinmailong Injection is a multicomponent pharmaceutical preparation whose active ingredients can act on multiple targets of the body through different routes. Clinical studies have shown that Xinmailong Injection can effectively treat a variety of cardiovascular system related diseases, such as pulmonary heart disease (PHD) [50, 51], ischemic cardiomyopathy (IHD) [52], dilated cardiomyopathy (DCM) [53], cardiogenic shock [54], and heart and kidney syndrome [55, 56], etc. Due to the lack of basic research in related fields, the intervention mechanism of Xinmailong Injection is not yet clear. More and more tissue cytology research, molecular biology research, and genetic research are being expected. To study the pharmacological mechanism of Xinmailong Injection, it is necessary to study not only the single active ingredients but also the common pathways, regulatory factors, and targets of the active ingredients in the process of initiation, so as to construct a complete system of intervention mechanisms. This will provide a reference for the formulation of more reasonable clinical prescriptions for Xinmailong Injection and further research and development of new drugs.

## 5. Conclusion

Xinmailong Injection can protect cardiomyocytes and maintain the normal function of the heart in various ways, thus effectively preventing the development of cardiovascular disease. Therefore, Xinmailong Injection has great potential for clinical application, and more basic researches need to be carried out to explore the medicinal value of Xinmailong Injection.

## Figures and Tables

**Table 1:** Search Strategy in PubMed.

Search	Query	Items found
#1	Search *Periplaneta Americana*[MeSH Terms]	1191
#2	Search *Periplaneta Americana*[Title/Abstract]	1688
#3	Search American cockroach[MeSH Terms]	1191
#4	Search American cockroach[Title/Abstract]	592
#5	Search Xinmailong[Title/Abstract]	12
#6	#1 or #2 or #3 or #4 or #5	2212
#7	Search heart failure[MeSH Terms]	112410
#8	Search heart failure[Title/Abstract]	158098
#9	Search ventricular dysfunction[Title/Abstract]	16291
#10	Search cardiovascular[MeSH Terms]	1192914
#11	Search cardiovascular[Title/Abstract]	410595
#12	Search heart[Title/Abstract]	793341
#13	Search cardiac[Title/Abstract]	568823
#14	Search myocardium[Title/Abstract]	71660
#15	Search myocardial[Title/Abstract]	322958
#16	Search blood vessel[Title/Abstract]	18964
#17	Search vascular[Title/Abstract]	549712
#18	Search artery[Title/Abstract]	488798
#19	#7 or #8 or #9 or #10 or #11 or #12 or #13 or #14 or #15 or #16 or #17 or #18	2758422
#20	#6 and #19	81

**Table 2:** Basic information on the included studies.

Research	Experimental model	Observation indicators	Effect of XMLI
Wu et al. 1995[10]	rabbits with myocardial injury induced by isoproterenol	J-point displacement (increased or lowered) on the ECG; frequency of ischemic arrhythmia	After pretreatment with XMLI, the J-point displacement on the ECG caused by isoproterenol can be significantly reduced, so that the J-point position is close to the normal equipotential line. And the frequency of ischemic arrhythmia can be significantly reduced.

Wu et al. 2002[11]	rats with myocardial injury induced by isoproterenol	J-point displacement (increased or decreased) on the ECG; CPK, LDH in serum; MDA, SOD, GSH-PX in myocardial tissue	After pretreatment with XMLI, the J-point displacement on the ECG caused by isoproterenol can be significantly reduced, MDA level be reduced, CPK and LDH activity be reduced, GSH-PX and SOD activity be increased.

Tian and Yang 2010[12]	rabbits with ischemia-reperfusion induced by hypovolemic shock	SOD; NO^−2^/NO^−3^; blood pH; urine output	XMLI can increase SOD activity and NO_2_^−^/NO_3_^−^ level, correct acidosis, increase blood pH, improve circulating blood flow, and increase urine output.

Li et al. 2017[13]	isolated rat heart; H9C2 cells (rat cardiomyocytes)	cardiac function; [Ca^2+^]i of H9C2 under electrical stimulation; Ca^2+^ influx; intracellular Ca^2+^ store; T-type Ca^2+^ channels; NCX; Na^+^/K^+^-ATPase activity; ROS; SOD-1; SOD-2; HO-1	XMLI can increase intracellular Ca^2+^ level by activating T-type Ca^2+^ channels and inhibiting Na^+^/K^+^-ATPase. XMLI can also reduce the production of ROS and enhance the expressions of SOD-1, SOD-2, HO-1.

Cao et al. 2009[14]	young rabbits with myocardial ischemia-reperfusion injury	CPK; iNOS; ecNOS; MDA	XMLI can reduce the ecNOS, MDA, CK activity and inflammatory cell infiltration in the reperfusion injury model.

Zhang et al. 2011[15]	asphyxiating newborn rats	CK-MB; NF-*κ*B; TNF-*α*	After 6 hours of asphyxia in neonatal rats, serum CK-MB level, myocardial NF-*κ*B, and TNF-*α* expressions peaked. XMLI can significantly reduce serum CK-MB level, myocardial NF-*κ*B, and TNF-*α* expressions.

Liu et al. 2014[16]	healthy rats for preparing doxorubicin-induced HF model	LVEF; LVFS; SOD; MDA; BNP; cTnI	XMLI can reduce doxorubicin-induced cardiotoxicity, increase SOD activity and cardiac EF, and decrease MDA activity.

Liu et al. 2016[17]	healthy rats for preparing doxorubicin-induced HF model	LVEF; LVFS; SOD; MDA; BNP	XMLI can reduce doxorubicin-induced cardiotoxicity, increase SOD activity and cardiac EF, and decrease MDA activity.

Duan et al. 2018[18]	healthy rats for preparing epirubicin-induced HF model	body weight; LVEF; SOD; MDA; BNP; cTnI	XMLI can reduce epirubicin-induced cardiotoxicity, increase SOD activity and EF, decrease MDA activity and cTnI level, and prevent weight loss.

Yuan and Jiang 2017[19]	H9C2 cells (rat cardiomyocytes)	cardiomyocyte activity; SOD; MDA; Caspase-3 activity; autophagy-associated protein LC3B activity	XMLI can reduce doxorubicin-induced cardiotoxicity, increase cell activity and SOD activity, and decrease MDA level, Caspase-3 activity, and protein LC3B accumulation.

Li et al. 2016[20]	healthy rats for preparing epirubicin-induced HF model	cardiac function; survival rate; body weight; LVPW thickness; EF; ECG; accumulation of collagen; expression of MMPs and TIMP4; expression of TGF-*β*1 mRNAs; expression of Ace, Ace2, Mas, and Agtr1 mRNAs; autophagy; expression of PI3K and AKT; the phosphorylation of P38 MAPK and ERK1/2	XMLI can enhance the survival rate of rats from epirubicin-induced HF. XMLI can prevent LV dilatation, improve cardiac function. And the treatment of the epirubicin rats with XMLI significantly recovered these changes, such as QT, QTc intervals and QRS duration. Furthermore, XMLI can significantly inhibit the accumulation of collagen, reduce the MMP9 and TGF-*β*1. XMLI can also decrease Beclin1 and Atg7, activate the PI3K/AKT signaling pathway and inhibit the ERK1/2 and P38 MAPK signaling pathways.

Li et al. 2019[21]	H9C2 cells (rat cardiomyocytes)	IL-1b; IL-6; TNF-*α*; expression of LC3, PINK1, Parkin, Nix, Beclin-1; Mitofusin1, Mitofusin2, Opa1, Drp1 and P62	XMLI can increase cell viability and the release of LC3 in H9C2 cells. XMLI can reduce the level of cTnI, CK-MB, IL-1b, IL-6, and TNF-*α*. XMLI can increase the protein and mRNA expression of PINK1, Parkin, Nix, Beclin-1 and decrease expression of Mitofusin1, Mitofusin2, Opa1, Drp1, and P62.

Peng et al. 2002[22]	cardiomyocytes of healthy rats	intracellular Ca^2+^ store	0.19, 0.38 and 0.76g/L XMLI can increase the content of Ca^2+^ in cardiomyocytes. This effect of 0.38g/L XMLI can be slightly inhibited by 40*μ*mol/L verapamil (inhibition rate is 20%), however, this effect of 0.19 and 0.76g/L XMLI cannot be inhibited by 40*μ*mol/L verapamil.

Peng et al. 2003[23]	hypoxia-reoxygenated cardiomyocytes of rats	cardiomyocyte [Ca^2+^]i; MDA; SOD	XMLI can increase [Ca^2+^]i in hypoxia and hypoxia-reoxygenated myocardium, increase SOD level and decrease MDA level. And this effect cannot be inhibited by verapamil.

Liu et al. 2017[24]	alcoholic cardiomyopathy rats	myocardial tissue microstructure changes; indicators of cardiac Doppler ultrasound; CTGF	XMLI can reduce CTGF expression and ECM collagen deposition in rats with alcoholic cardiomyopathy.

Qi et al. 2017[3]	HF mice with TAC surgery; H9C2 cells (rat cardiomyocytes)	LVPW thickness; EF; FS; phosphorylation of ERK1/2, AKT, and GSK3*β*; expression of GATA4 in the nucleus	XMLI can reduce the diastolic thickness of the LVPW, increase EF and FS. XMLI can inhibit the phosphorylation of ERK1/2, AKT, and GSK3*β*, subsequently inhibiting protein expression of GATA4 in the nucleus.

Wu et al. 2009[25]	pulmonary hypertension rats	mean PAP; ET; NO	XMLI can significantly reduce PAP while increasing plasma NO level and lowering plasma ET level.

Huang and Wu 2009[26]	asphyxiating newborn rats	CK; ET-1; HIF-1*α*; myocardial histopathological changes	XMLI can reduce the expression of HIF-1*α*, reduce plasma ET-1 level, and alleviate myocardial hypoxia-ischemic injury.

Zhang et al. 2012[27]	AS rats	NO; ET; PGI2; TXA2	XMLI can significantly increase serum NO level and plasma PGI2, and significantly reduce plasma ET level in AS rats.

Sun and Zhang 2012[28]	AS rats	TC; TG; LDL-C; HDL-C	XMLI can significantly reduce the levels of serum TC, TG and LDL-C and increase the level of HDL-C in AS rats.

Wu et al. 2002[29]	healthy rats for preparing arrhythmia model	survival time; the lethal dose of BaCI_2_ in rabbits; the rate of sinus rhythm conversion	XMLI can significantly increase the lethal dose of BaCI2 in rabbits and prolong the survival time of rabbits. It prevents and reduces VPB, VT, and VF caused by BaCI2. It can also significantly improve the rate of sinus rhythm conversion.

Li et al. 2018[30]	healthy rabbits for preparing atrial electrical remodeling model	AERP	XMLI can prevent the shortening of AERP200 and AERP150 after 6 hours of rapid pacing.

XMLI: Xinmailong Injection; ECG: electrocardiogram; CPK: creatine phosphokinase; LDH: lactate dehydrogenase; MDA: malondialdehyde; SOD: superoxide dismutase; GSH-PX: glutathione peroxidase; NO_2_^−^/NO_3_^−^: ratio of nitrite to nitrate; NCX: Na^+^/Ca^2+^ exchanger; ROS: reactive oxygen species; HO: heme oxygenase; iNOS: inducible nitric oxide synthase; ecNOS: endothelial constitutive nitric oxide synthase; CK-MB: creatine kinase-MB; NF-*κ*B; nuclear factor-kappa B; TNF-*α*: tumor necrosis factor-*α*; HF: heart failure; LV: left ventricular; EF: ejection fraction; FS: fraction shortening; BNP: brain natriuretic peptide; cTnI: cardiac troponin I; LVPW: left ventricular posterior wall; MMP: matrix metalloproteinase; TIMPs: tissue inhibitors of matrix metalloproteinase; TGF-*β*1: transforming growth factor-*β*1; Ace: angiotensin-converting enzyme; Mas: proto-oncogene Mas; PI3K: phosphatidylinositol 3 kinase; AKT: protein kinase B; GSK3*β*: glycogen synthase kinase 3*β*; MAPK: mitogen-activated protein kinases; ERK1/2: extracellular regulated protein kinases 1/2; Atg7: autophagy-related gene 7; QTc: corrected QT; IL: interleukin; CTGF: connective tissue growth factor; ECM: extracellular matrix; TAC: transverse aortic coarctation; PAP: pulmonary artery pressure; ET: endothelin; NO: nitric oxide; HIF-1*α*: hypoxia-inducible factor-1*α*; AS: atherosclerosis; PGI2: prostacyclin 2; TXA2: thromboxane 2; TC: total cholesterol; TG: triglyceride; LDL-C: low density lipoprotein cholesterol; HDL-C: high density lipoprotein cholesterol; VPB: ventricular premature beat; VT: ventricular tachycardia; VF: ventricular fibrillation; AERP: atrial effective refractory period.
